# Pooling strategy and chromosome painting characterize a living zebroid for the first time

**DOI:** 10.1371/journal.pone.0180158

**Published:** 2017-07-12

**Authors:** Alessandra Iannuzzi, Jorge Pereira, Clara Iannuzzi, Beiyuan Fu, Malcolm Ferguson-Smith

**Affiliations:** 1 Laboratory of Animal Cytogenetics and Genomics, National Research Council of Italy, Institute of Animal Production Systems in Mediterranean Environments (ISPAAM), Naples, Italy; 2 Cytocell Ltd., Cambridge Technopark, Cambridge, United Kingdom; 3 Department of Biochemistry, Biophysics and General Pathology, Università degli Studi della Campania “Luigi Vanvitelli”, Naples, Italy; 4 Cytogenetic Facility, Wellcome Trust Sanger Institute, Hinxton, United Kingdom; 5 Cambridge Resource Centre for Comparative Genomics, University of Cambridge Department of Veterinary Medicine, Cambridge, United Kingdom; Fralin Life Science Institute, Virginia Tech, UNITED STATES

## Abstract

We have investigated the complex karyotype of a living zebra-donkey hybrid for the first time using chromosome-specific painting probes produced from flow-sorted chromosomes from a zebra (*Equus burchelli*) and horse (*Equus caballus*). As the chromosomes proved difficult to distinguish from one another, a successful new strategy was devised to resolve the difficulty and characterize each chromosome. This was based on selecting five panels of whole chromosome painting probes that could differentiate zebra and donkey chromosomes by labelling the probes with either FITC or Cy3 fluorochromes. Each panel was hybridized sequentially to the same G-Q-banded metaphases and the results combined so that every zebra and donkey chromosome in each suitable metaphase could be identified. A diploid number of 2n = 53, XY was found, containing haploid sets of 22 chromosomes from the zebra and 31 chromosomes from the donkey, without evidence of chromosome rearrangement. This new strategy, developed for the first time, may have several applications in the resolution of other complex hybrid karyotypes and chromosomal aberrations.

## Introduction

In recent times animal breeders and zoos have created, either by accident or design, a spectacular range of mammalian hybrids, from Grizzly-Polar bear, Coywolf, Savannah cat and Liger (lion-tiger), to Zebroids [1–2], Dzo [3] (cattle-Yak), Beefalo [4] (American bison and cattle), geep [5–6–7] (sheep-goat) and Cama (camel-llama). Most of these hybrids are listed in Annie Gray's masterful bibliography [8] and they represent exciting and new scientific challenges for today's molecular and developmental biologists. In the past, numerous successful attempts at breeding have been made but the hybrids are sterile in all cases [9] with the occasional exception of mules [10] (horse-donkey). Up to now, most of these hybrids have been studied using conventional cytogenetics, with no banding results and no information about chromosomal rearrangements.

Chromosomal rearrangements contribute to the evolution and maintenance of species barriers via two primary mechanisms. First, by hybrid sterility that arises from the heterozygous rearrangements [9] that disrupt meiotic pairing, or from the production of chromosomally unbalanced gametes [11–12]. Second, by the suppression of recombination in rearranged chromosomal regions that consolidate existing barriers and promote adaptive divergence and speciation in the face of gene flow [13]. Historically, chromosomal rearrangements have been considered a major source of hybrid sterility, particularly in plants [11], but this view has fallen from favour as a mechanism of postzygotic reproductive isolation. In sterile hybrids the main problems are difficulty in synapsis during meiosis that may lead to non-homologous recombination and maturation arrest during gametogenesis [9].

There are several reports of chromosome studies on zebroids including zebra hybrid [14], donkey-grevy zebra hybrid [2], zebra-donkey [1] and some others reported by Gray [8]. These cases were studied using conventional cytogenetics that provided little information on possible chromosomal rearrangements. Nowdays, four zebra-donkey living hybrids exist in different world areas: Italy (male foaled by a donkey), Mexico (male foaled by a zebra), China (female foaled by a zebra) and Georgia (female foaled by a donkey). They were born after a natural mating and present a similar phenotype with distinctive black stripes of zebra on the legs and ears and the narrow head of a donkey; furthermore, there are no scientific data about their genetic and cytogenetic characterization.

**Fig 1:**
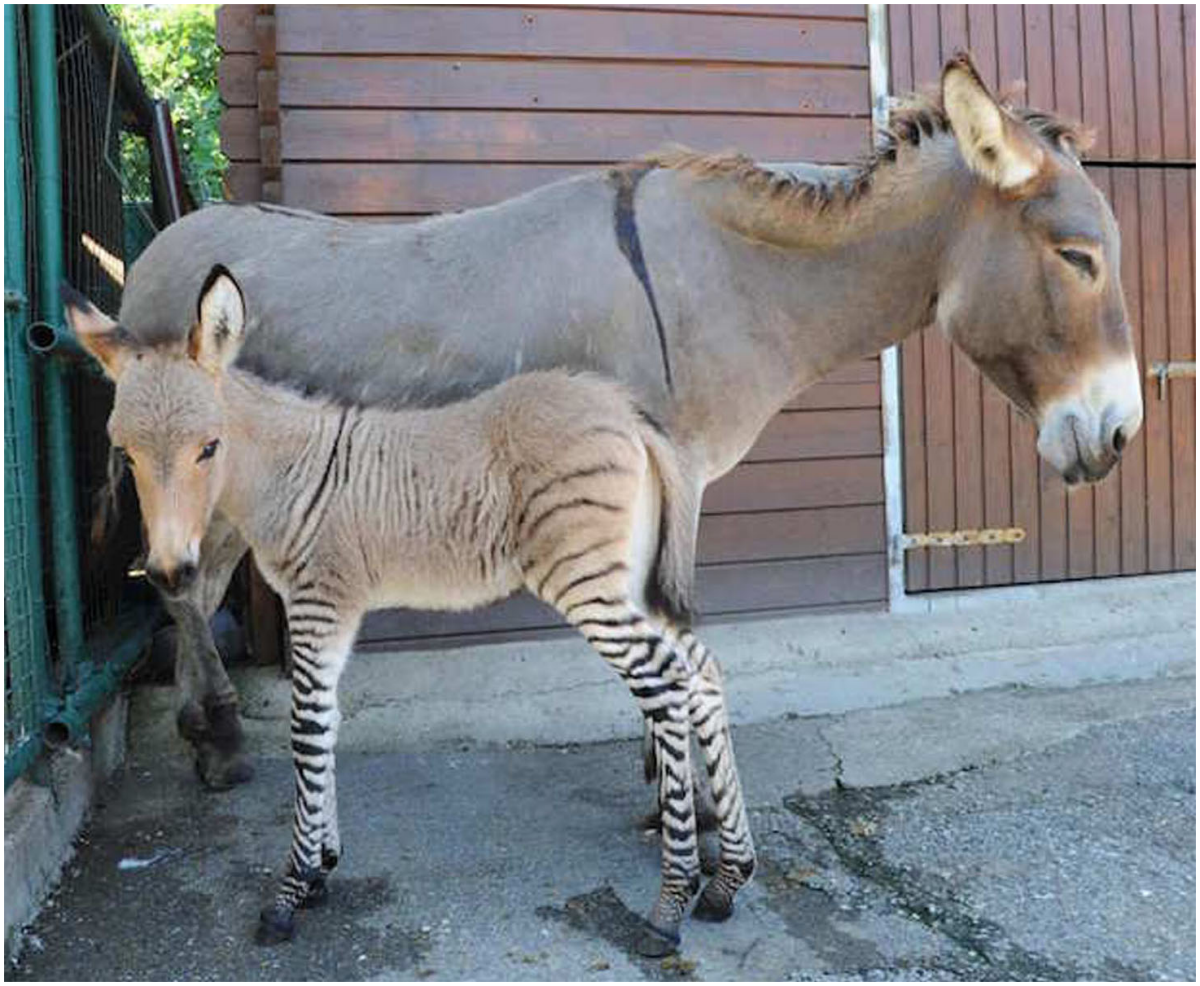
Zebroid. The newborn zebroid (2n = 53, XY) on the left, and his mother donkey (*Equus asinus*, 2n = 62, XX) (Courtesy of A. Massimo).

In this study we report the complete chromosomal characterization about one of zebroids mentioned above, using chromosome painting, pooling strategy and sequential Multicolour fluorescence in situ hybridization (M-FISH) for the first time in a hybrid. The zebroid male was foaled by a donkey (Equus asinus, 2n = 62, XX) after a natural mating with a zebra sire (Equus burchelli, 2n = 44, XY) in an Italian animal rescue centre close to Florence in July 2013. The owner stated that zebra and donkey were separated from each other and according to Dr. Luca Moretti (veterinary in charge of the centre) the hybrid appeared healthy and without obvious malformation (Fig 1). The zebroid was raised by his mother, sharing the same housing and he has received both medical care and suitable diet by the veterinary of the centre. When the decision to investigate was made, our aim was to determine the karyotype and look for evidence of chromosome rearrangements. We asked to the owner of the zebroid the permission to conduct cytogenetic investigations to the animal and, after his approval, Dr. Moretti performed the blood collection avoiding any pain or distress to the animal during the process.

## Materials and methods

### Ethical approval

This study was performed in strict accordance with the recommendations in the Guide for the Care and Use of Laboratory Animals of the National Institutes of Health. The protocol was approved by the Committee on the Ethics of Animal Experiments of the “Institute of Animal Production Systems in Mediterranean Environments (ISPAAM)” of Naples, National Research Council (CNR) of Italy, (Permit Number: 1591/2014).

### Nomenclature

The *Equus asinus* (EAS) chromosomes and idiogram were identified according to the standard karyotype of the donkey [15]. The *Equus burchelli* (EBU) chromosomes and idiogram were identified according to Yang et al. [16] and Hansen et al. [17]. Chromosomes homologous between EBU-ECA-EAS were identified following Musilova et al. [18].

### Metaphase preparations

Zebroid metaphases were obtained from peripheral blood sample cultured in RPMI-1640 (1X) medium enriched with fetal calf serum (10%), antibiotic-antimycotic mixture (penicillin-streptomycin, 1%), L-glutamine (1%), Concanavalin A (15 μg/ml) and finally incubated for 72 h at 37,7°C. Two types of cell cultures were made, either without adding any base analog (normal cultures), or with BrdU (5-bromo-2'-deoxyuridine) and MTX (Methotrexate) to obtain G-Q-banding patterns by early incorporation of BrdU and cell synchronization with MTX. BrdU (20 μg/ml) and MTX (0.5 μg/ml) were added 24 h before the end culture. After 17 h the cells treated with BrdU and MTX were washed in sterile Puck's saline and allowed to recover for 6 h in fresh medium with thymidine (7 μg/ml) as described in Iannuzzi et al. [19–20]. The colcemid treatment (0.1 μg /ml) was performed in the last hour to all cell cultures. Chromosome preparations were obtained by hypotonic treatment (KCl 0.066M not buffered) at 37°C for 20 minutes. Finally, cells were fixed in methanol: acetic acid (3:1) with three successive changes of fixative solution. Further details can be found in Iannuzzi and Di Berardino [21].

### Flow sorting and generation of chromosome-specific paint probes

Chromosome suspensions of EBU and ECA were sorted on a dual laser cell sorter (FACStar Plus, Becton Dickinson) at the Cambridge Resource Centre for Comparative Genomics and chromosome-specific painting probes were made by degenerate oligonucleotide PCR amplification of flow-sorted chromosomes as described for these two species previously [16–22]. The probes were labelled in a secondary amplification step with either biotin-16-dUTP (Roche) or directly with Cy3-dUTP (Amersham).

### Pooling strategy and Sequential Multicolour Fluorescence *in situ* Hybridization

For the M-FISH technique slides allocated for G-Q-banding were baked at 65°C for 2 h, denatured in a 70% formamide/ 2XSSC solution at 65°C for 2 min and dehydrated through a 70, 90 and 100% glacial ethanol series. Each chromosome-specific probe was selected for inclusion in one of five probe pools (Table 1) based on criteria (banding, size and centromere position) that produced the best distinction between chromosomes hybridized by the probe pool. Each pool was denatured at 65°C for 10 min and then pre-annealed by incubation at 37°C for 30–60 min. The pre-annealed pool was applied to the slide, covered with 22-mm coverslips, sealed with rubber cement and incubated for 24 h at 37°C. Post-hybridization washes involved two series of 5-min incubations in 50% formamide/50% 2XSSC at 45°C followed by two series of 5-min incubations in 2XSSC at 45°C. The biotin was detected with FITC-conjugated avidin, incubating the slide in moisture chamber in dark at 37°C for 1h. The hybridization, post-hybridization washes and detection conditions follow the procedure described by Yang et al. [16]. After detection, slides were mounted in Vectashield mounting medium with DAPI (4’,6-diamidino-2-phenylindole, Vector Laboratories). The coverslip was removed after each M-FISH analysis and the slide was washed in a 4xT solution (20% 20xSSC, 0.05% Triton-X100) rinsed and dried before starting a new hybridization with the next probe pool, as described by Pauciullo et al. [23].

**Table 1:** Painting probes pools. Composition of the five painting pools used in sequential M-FISH on zebroid metaphases showing each probe and its fluorophore.

WCPs	Fluorophore
**1**^**st**^ **pool**	
EBU 8+X	FITC
ECA X	CY3
EBU 10+12	CY3
ECA 10	FITC
ECA Y	FITC
**2**^**nd**^ **pool**	
EBU 9	FITC
EBU 18	FITC
EBU 5	FITC
ECA 14	CY3
EBU 16	CY3
EBU 21	CY3
**3**^**rd**^ **pool**	
EBU 13+14	FITC
EBU 15	CY3
EBU 11	FITC
ECA 10p	CY3
EBU 2	FITC+CY3
**4**^**th**^ **pool**	
EBU 1	FITC
ECA 16+25	CY3
EBU 6	CY3
EBU 7	FITC
**5**^**th**^ **pool**	
EBU 3	FITC
EBU 4	CY3
EBU 19+20	CY3
EBU 19	FITC
EBU 17	CY3

### Microscopic analysis

The images were captured, after each hybridization, by using a Leica DMRXA fluorescence microscope equipped with 100x lens, DAPI, FITC, Texas Red specific filters and Photometrics Sensys camera. At least 30 metaphases were acquired and processed using Leica QFISH software (Leica Microsystems). Digital images were captured in grey-scale and false colours were created by the image-analyzing systems for an evaluation of the probes.

## Results

**Fig 2:**
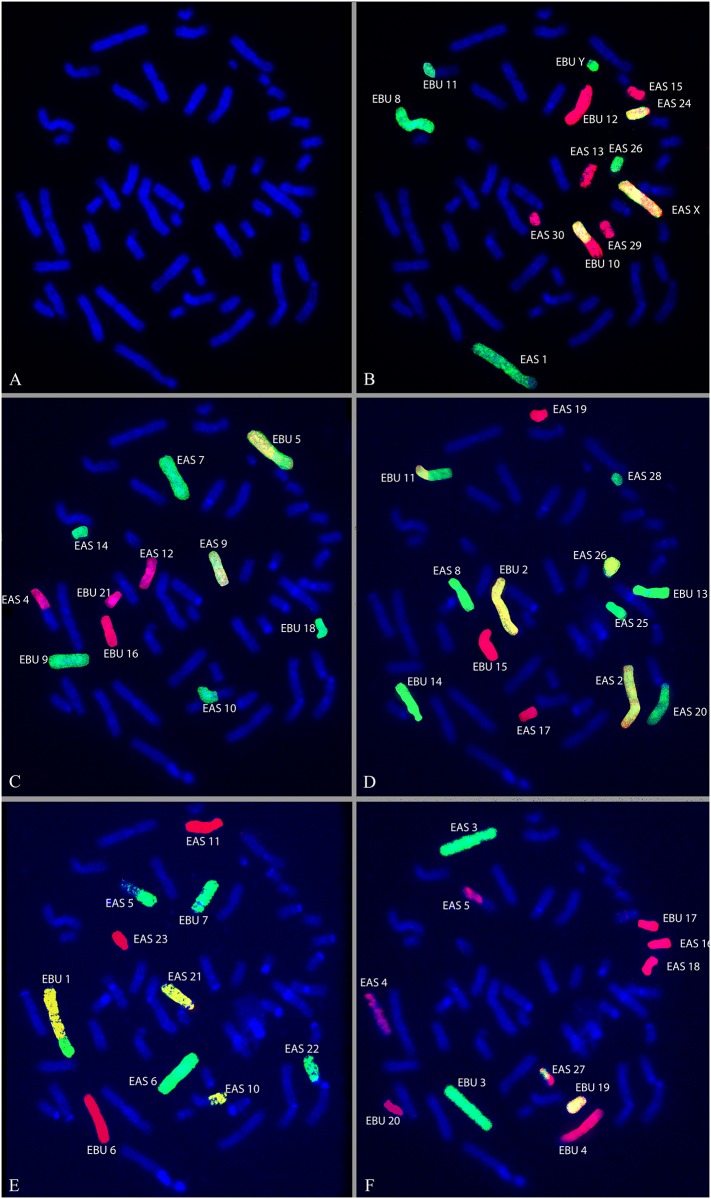
Sequential M-FISH on G-Q banded zebroid of metaphase chromosomes. (A) DAPI G-Q-banded metaphase of the zebroid. (B-F) Same metaphase following five sequential M-FISH with the pooled probes showing the identification of each EBU and EAS chromosome. Scale bar = 10 μm.

**Fig 3:**
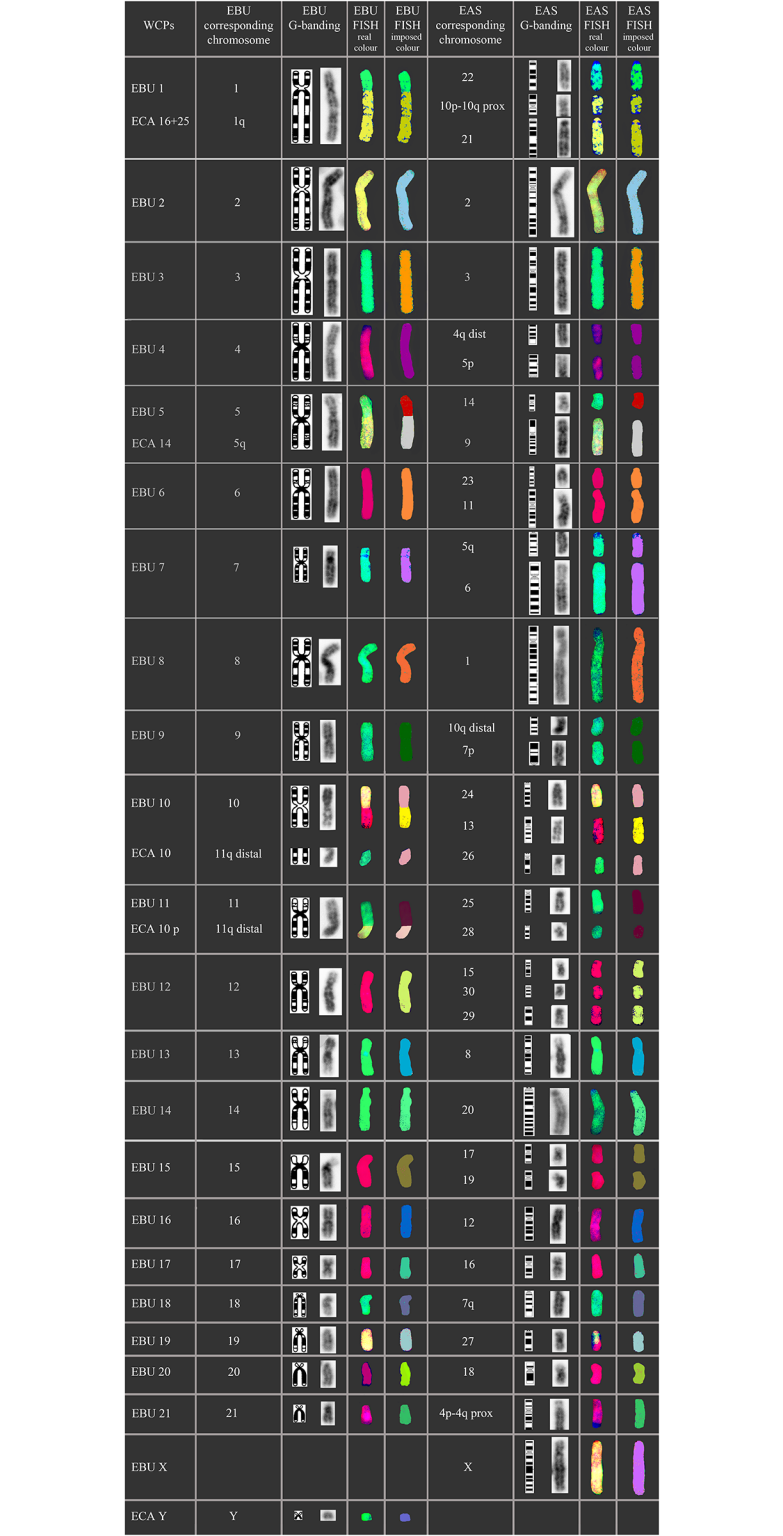
Identification of zebroid metaphase chromosomes. Plan showing zebroid chromosome identification. From the left, WCPs from EBU and ECA used for FISH; EBU regions hybridized; EBU idiogram; EBU DAPI inverted image; EBU painted chromosome showing real and imposed image; region of homology with EAS; EAS idiogram; EAS regions hybridized by EBU paint showing real and imposed image.

**Fig 4:**
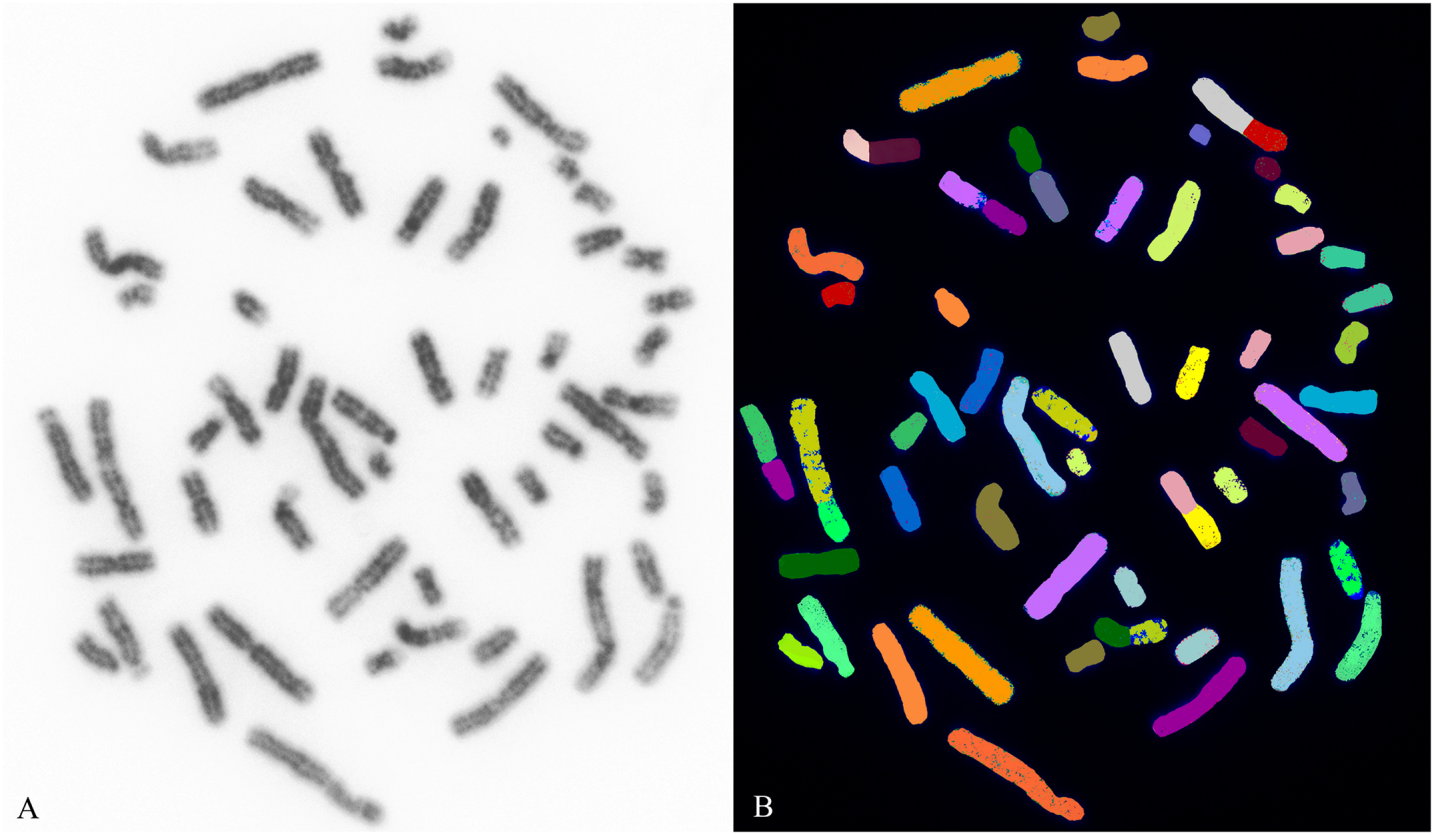
Zebroid G-Q banded and M-FISH of metaphase chromosomes. The same metaphase shown in Fig 2 demonstrates that all zebroid chromosomes can be identified by the imposed colours indicated in Fig 3. Scale bar = 10 μm.

Chromosome analysis of the somatic cells of the hybrid reveals a 2n = 53, XY karyotype and a FN = 95 (chromosome arms) (S1 Fig). Sequential M-FISH, using the five chromosome painting panels with EBU and ECA WCPs (Whole chromosome painting probes), shows an entire haploid set of 22 chromosomes of EBU in the zebroid. This was achieved by identifying and excluding the 32 chromosomes of ECA by an analysis of EBU homology on EAS chromosomes, together with an analysis of the size and DAPI (4',6-diamidino-2-phenylindole) banding patterns of EBU and EAS chromosomes from published karyotypes (see Nomenclature). The chromosome painting results from each of the five probe panels listed in Table 1 together reveal the identity of all chromosomes in the same metaphase and this is illustrated in Fig 2. The hybridizations with horse chromosome-specific probes ECA10p, 14, 16+25 and Y, help to identify EAS 26, 9, 10p-10qprox, 21 and the Y chromosome respectively. Fig 3 shows details of the results for each WCP used: the EBU and corresponding EAS chromosome; the EBU and EAS G-Q-banded idiogram and the corresponding DAPI-inverted chromosome; the M-FISH results showing EAS and EBU chromosomes with real and false (imposed) colour images after digital processing. The EBU probes reveal chromosome fusions and fissions that have occurred during the divergence of the two species and indicate the regions of EBU homology on EAS chromosomes that allow the identification of EAS chromosomes. The colours imposed on the chromosomes in Fig 3 are used on the same metaphase in Fig 4 to confirm that all chromosomes are accounted for in the hybrid. It is apparent from these findings that the zebroid karyotype contains a haploid set of chromosomes derived from each of its parents without evidence of chromosome rearrangement. This data based on the evaluation of at least 30 different metaphases that have showed the same set of chromosomes demonstrated with M-FISH analisys.

## Discussion

Since the first manuscript on M-FISH [24], several changes or modifications were introduced in microscopy, image analysis and probe set generation to optimize the method and to get the best results. Furthermore, the generation of the very complex probe set was facilitated by the introduction of a "pooling strategy" [25–26] where the number of labeling reactions is reduced to the number of fluorochromes used. In addition, FISH-based banding technologies, such as cross-species colour banding [27] or high resolution multicolor-banding [28] were developed for karyotyping and to facilitate the identification of intrachromosomal rearrangements. In this case, the pairing of homologous chromosomes in the zebroid proved difficult as the chromosome count gave a diploid number of 53 and different chromosomes were derived from each parent, presumably 22 from the zebra and 31 from the donkey. For this reason, we have conceived a new strategy combining the above mentioned techniques with a technique developed by us. In fact, five sequential hybridizations were made on the same slide in a series of experiments using five different pools (Table 1) composed of probes from different zebra and horse chromosomes, in order to identify all chromosomes in the same metaphase. The acquired images were then analysed enabling individual EBU and EAS chromosomes to be classified based on size, centromere position, DAPI-banding and fluorochrome-labelled signal. Finally, digitally acquired and processed images revealed the identity of all chromosomes in a single metaphase (Fig 4). The pooling strategy designed to optimize the use of only two fluorochromes, generating three colours (red, green and yellow), for the chromosomes that cannot be identified easily from one another by size, centromere position and banding, has been the key to resolve the problem of chromosome identification in this rare zebroid. An additional advantage is that the procedure avoids the complicate and expensive approach of developing an individual M-FISH probe set for each species. This appears to be the first time such a pooling strategy has been used to evaluate a zebroid. Furthermore, the method may have application in the study of other complex hybrid karyotypes and chromosomal aberrations. We can conclude confidently from the unambiguous identification of all chromosomes that the zebroid has received a precise haploid set of chromosomes from each parent without rearrangement. Finally, this study would assume the sterility of the zebroid due to the very complex chromosomal organization; for this reason, we are planning to study sperm chromosome segregation upon reaching sexual maturity.

## Supporting information

S1 Fig Zebroid RBA and CBA of metaphase chromosomes. Sequential RBA (Acridine orange R banding)—CBA (Acridine orange C banding) technique showing the EBU Y and ECA 1 chromosomes. Scale bar = 10 μm.
(TIF)
