# Supplementary figures and images for: Pooling strategy and chromosome painting characterize a living zebroid for the first time
Source: PLoS One. 2017 Jul 12;12(7):e0180158. doi: 10.1371/journal.pone.0180158 (PMC5507506; doi:10.1371/journal.pone.0180158)

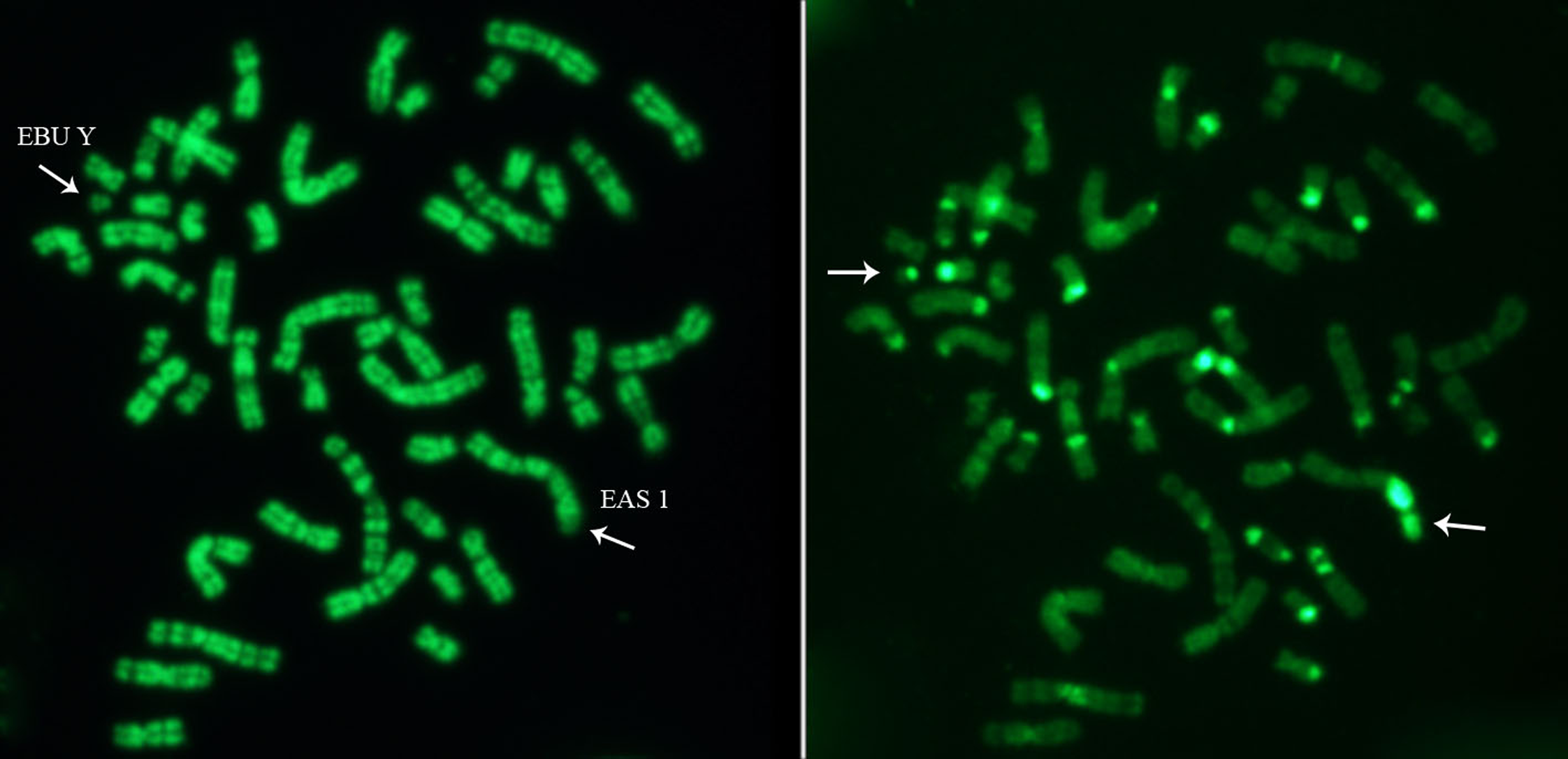

Supplement: S1 Fig — Sequential RBA (Acridine orange R banding)—CBA (Acridine orange C banding) technique showing the EBU Y and ECA 1 chromosomes. Scale bar = 10 μm. (TIF) [file pone.0180158.s001.tif]
